# Inference of person-to-person transmission of COVID-19 reveals hidden super-spreading events during the early outbreak phase

**DOI:** 10.1038/s41467-020-18836-4

**Published:** 2020-10-06

**Authors:** Liang Wang, Xavier Didelot, Jing Yang, Gary Wong, Yi Shi, Wenjun Liu, George F. Gao, Yuhai Bi

**Affiliations:** 1 CAS Key Laboratory of Pathogenic Microbiology and Immunology, Institute of Microbiology, Center for Influenza Research and Early-warning (CASCIRE), CAS-TWAS Center of Excellence for Emerging Infectious Diseases (CEEID), Chinese Academy of Sciences, Beijing, 100101 China; 2 School of Life Sciences and Department of Statistics, University of Warwick, Coventry, CV4 7AL UK; 3 Institut Pasteur of Shanghai, Chinese Academy of Sciences, Shanghai, 200031 China; 4 Département de microbiologie-infectiologie et d’immunologie, Université Laval, Québec City, QC G1V 0A6 Canada; 5 University of Chinese Academy of Sciences, Beijing, 101408 China; 6 Shenzhen Key Laboratory of Pathogen and Immunity, Second Hospital Affiliated to Southern University of Science and Technology, Shenzhen Third People’s Hospital, Shenzhen, 518112 China

**Keywords:** Phylogenomics, SARS-CoV-2, Viral infection, Epidemiology

## Abstract

Coronavirus disease 2019 (COVID-19) was first identified in late 2019 in Wuhan, Hubei Province, China and spread globally in months, sparking worldwide concern. However, it is unclear whether super-spreading events occurred during the early outbreak phase, as has been observed for other emerging viruses. Here, we analyse 208 publicly available SARS-CoV-2 genome sequences collected during the early outbreak phase. We combine phylogenetic analysis with Bayesian inference under an epidemiological model to trace person-to-person transmission. The dispersion parameter of the offspring distribution in the inferred transmission chain was estimated to be 0.23 (95% CI: 0.13–0.38), indicating there are individuals who directly infected a disproportionately large number of people. Our results showed that super-spreading events played an important role in the early stage of the COVID-19 outbreak.

## Abstract (web summary)

Although SARS-CoV-2 has spread rapidly, the contribution of super-spreading events to transmission is unclear. Here, the authors show that the number of secondary infections arising from an individual infection in the early phase of the outbreak was highly skewed, indicating that super-spreading events occurred.

## Introduction

Emerging and re-emerging pathogens have caused several outbreaks worldwide (such as influenza virus, Ebola virus, Zika virus, etc.), posing substantial threats to public health^1^. Six types of coronaviruses have previously been reported to infect humans, namely 229E, OC43, NL63, HKU1, severe acute respiratory syndrome coronavirus (SARS-CoV), and Middle East respiratory syndrome coronavirus (MERS-CoV)^2^. At the end of 2019, a novel coronavirus disease (COVID-19)^3–5^ caused by SARS-CoV-2 (also known as 2019-nCoV or HCoV-19^6^) was first reported in Wuhan, Hubei Province, China. COVID-19 subsequently spread throughout China and was detected abroad within weeks. The World Health Organization (WHO) declared COVID-19 a Public Health Emergency of International Concern on 30 January 2020^7^. Within a month, the global risk level of the COVID-19 was raised from “high” to “very high“^8^. On 11 March 2020, COVID-19 was declared a pandemic by the WHO^9^. Until 5 July 2020, more than 11 million confirmed COVID-19 cases have been reported in 216 countries/territories/areas^8^. The global spread of COVID-19 has thoroughly taxed the ability of many medical systems to handle such a rapid increase in the number of cases within such a short amount of time.

Super-spreading events (SSEs) are an important phenomenon in the transmission of many diseases (such SARS-CoV, MERS-CoV, Ebola virus, etc.), in which certain individuals infect many others, compared to the basic reproduction number (*R*_0_, indicating the average number of secondary cases caused by a single infected individual in a susceptible population)^10^. Quick identification of SSEs during the early phase of a disease outbreak could provide a basis to tailor prevention and control policies to prevent spread on a larger scale. Current approaches for the identification of SSEs are mainly based on retrospective epidemiological studies. However, epidemiological contract tracing mainly relies on patient recall, which can result in false negatives. Therefore, other methods that do not rely on epidemiological tracing data to identify SSEs are needed. In 2005, Lloyd-Smith et al.^11^ proposed an “individual reproductive number” (denoted as *ν*), representing the number of secondary cases caused by a particular infected individual, which was drawn from a continuous probability distribution with mean *R*_0_. In this framework, specific SSEs are events from the right tail of the distribution of *ν* and propensity for SSEs can be identified by estimating the skewness of the distribution of *ν*.

Although some sporadic reports suggested that SSEs may have occurred under certain circumstances^12–14^, it is still unknown whether SSEs played a role during the early phase of the COVID-19 pandemic. In this study, we reconstruct a transmission tree of COVID-19 based on genomic data and Bayesian inference under an epidemiological model, and then infer parameters of the offspring distribution in this transmission tree. We also test the impact of uncertainty from phylogeny on our results. Our results demonstrate that SSEs occurred during the early phases of the COVID-19 pandemic. These findings provide an important basis for guiding the development of prevention and control policies, especially for countries at the early stages of the COVID-19 pandemic.

## Results

### Inference of transmission chains during the early phase of the COVID-19 outbreak

**Fig. 1:**
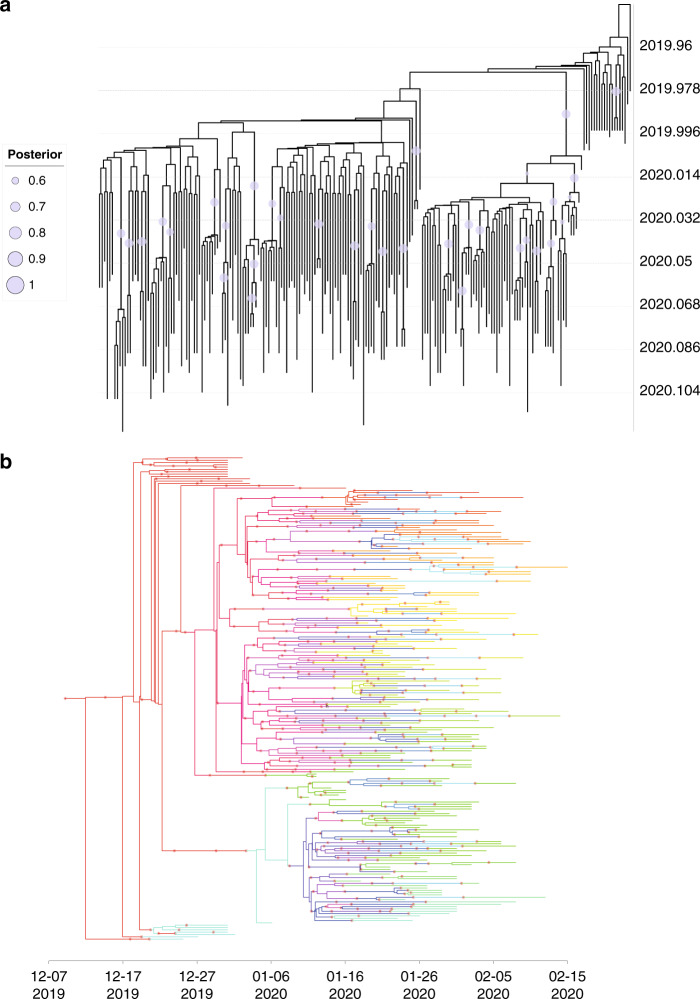
The phylogeny and transmission tree of SARS-CoV-2 during the early stage of COVID-19 outbreak. **a** Maximum clade credibility phylogeny estimated from genomic sequences of SARS-CoV-2 collected during the early stage of COVID-19 outbreak. Posterior probabilities >0.6 are shown with a purple circle. The size of the circle is proportional to the posterior probability. **b** Medoid transmission tree for the early stage of the COVID-19 pandemic. Patients are marked with different colors of branch on the phylogenetic tree. Red asterisks represent a transmission event.

We first constructed a dated phylogeny for SARS-CoV-2 during the early phase of the outbreak in China (Fig. 1a). Based on this dated phylogeny, the transmission tree was reconstructed and the medoid transmission tree is shown in Fig. 1b. There was considerable uncertainty in the inferred transmission tree, which is not shown in the medoid tree but is explored by the Markov Chain Monte Carlo (MCMC). To illustrate this uncertainty about who infected whom, we computed the probability of direct transmission from any case to any other. In total, we identified 18 pairs of patients with bidirectional probability for direct transmission (calculated by summing up the directed transmission probability in both directions) >0.5, indicating one of a pair of patients was directly infected by another (Supplementary Data 1). The number of intermediates in the transmission chains between each patient pair ranged between 0.002 (representing almost certain direct transmission) to 15.60, with a mean of 8.60 (Supplementary Data 2), indicating many patients have not been sampled in this transmission chain.

### Validation of inferred transmission tree

**Fig. 2:**
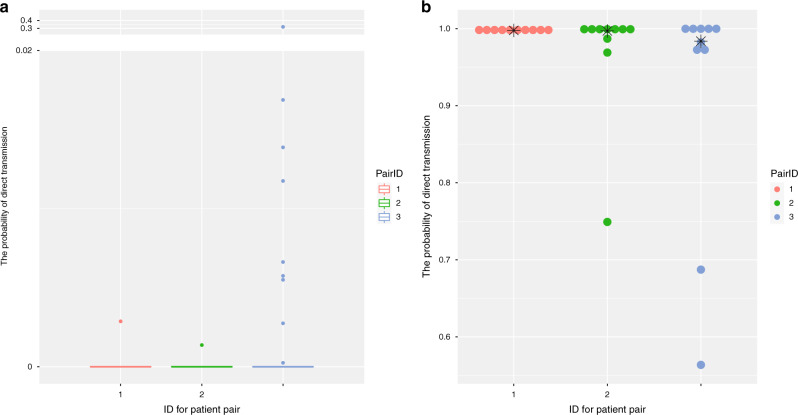
Validation of direct transmission events with high quality. **a** Boxplot of the bidirectional probability for direct transmission between three patients (ID:1, 2, and 3 represent EPI_ISL_408486, EPI_ISL_421236, and EPI_ISL_412898, respectively) and others (do not include the person who directly transmit to each other). Upper bound, center, and lower bound of box represent the 75th percentile, the 50th percentile (median), and the 25th percentile, respectively. Whiskers represent 1.5× interquartile range and points are outliers. **b** Dotplot of the bidirectional probability for direct transmission of three paired patients with high quality (ID:1, 2, and 3 represent EPI_ISL_408486 vs. EPI_ISL_421252, EPI_ISL_421236 vs. EPI_ISL_421235, and EPI_ISL_412898 vs. EPI_ISL_402127, respectively) using different phylogeny. The dot represents the result from randomly selected tree. The star represents the result from MCC tree.

We next verified the stability of the transmission tree by cross-validating the direct transmission events identified in our study. We removed one patient (i.e., EPI_ISL_421252, EPI_ISL_421235, and EPI_ISL_402127) from the top three direct transmission pairs (i.e., EPI_ISL_408486 vs. EPI_ISL_421252, EPI_ISL_421236 vs. EPI_ISL_421235, and EPI_ISL_412898 vs. EPI_ISL_402127) with the highest bidirectional probability for direct transmission in Supplementary Data 1, to generate a reduced dataset. We then repeated the same analysis. If the remaining patient from a directed transmission pair was inferred to be infected by an unsampled patient in this reduced dataset, it demonstrated that the inferred direct transmission event is reliable. We found that most probabilities of direct transmission between these three patients and others in this transmission tree were close to zero, and the highest possibility of directed transmission for these three individuals being infected by others are 0.0029, 0.0014, and 0.32, respectively (Fig. 2a). This result indicates that they were likely directly infected by an unsampled patient rather than those in this reduced dataset, providing further evidence that the identification of direct transmission events were likely to be reliable. We also evaluated whether the uncertainty on the phylogeny affected the result by independently performing analysis on ten randomly selected trees from MCMC chains. We found that all runs had unidirectional probability of direct transmission between each pair >0.5, for these three patient pairs (Fig. 2b), indicating the inference of direct transmission events were robust to the uncertainty of phylogeny. In summary, the transmission chains and directed transmission events inferred in our study were robust.

### Identification of SSEs

**Fig. 3:**
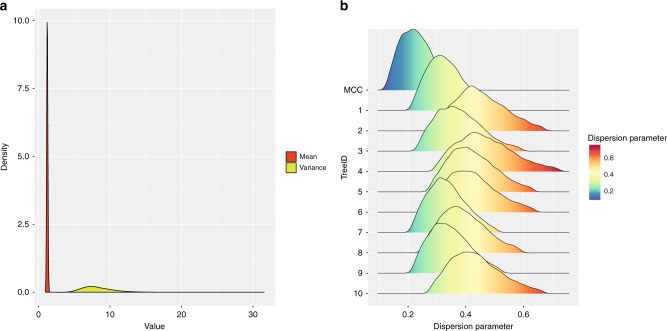
Heterogeneity of transmission during the early stage of COVID-19 outbreak. **a** The mean and variance of the offspring distribution along MCMC iterations. **b** The 95% CI distribution of dispersion parameter using MCC tree and ten randomly selected trees from the MCMC chains.

The offspring distribution (number of secondary infections caused by each case) was also inferred in this study. The offspring distribution was assumed to follow a negative binomial distribution and we computed its mean and variance at each MCMC step. The mean of the offspring distribution is the basic reproduction number *R*_0_ and was equal to 1.23 (95% confidence interval (CI): 1.09–1.39), indicating that on average an infected individual could cause 1.23 infections in a susceptible population. The variance for the offspring distribution was estimated as 8.31 (95% CI: 5.06–13.39). As shown in Fig. 3a, the variance was significantly larger than the mean, which is known as overdispersion. In addition, the dispersion parameter of the offspring distribution was estimated as 0.23 (95% CI: 0.13–0.39), further demonstrating the overdispersion in offspring distribution. We also tested how the uncertainty of phylogeny affected the estimation of the offspring distribution parameters. The dispersion parameter of the offspring distribution based on ten randomly selected trees was slightly higher than for the maximum clade credibility (MCC) tree (Fig. 3b). As the MCC tree is more accurate than to trees sampled in MCMC chains, this result suggested that the uncertainty of the phylogeny would cause an overestimation of the dispersion parameter of the offspring distribution, which meant that it would underestimate the importance of SSEs. In summary, the number of offspring inferred from the transmission tree was highly skewed, indicating SSEs did exist during the early stages of the COVID-19 pandemic.

## Discussion

Infectious diseases, such as COVID-19 and influenza, can spread globally at a very rapid rate due to globalization and increased international travel and trade. In addition, differences in the preparedness and vulnerability of different countries against COVID-19 will lead to different impacts on countries with imported cases^15^. SSEs, in which a high number of contacts are infected, have been identified for other diseases (such as SARS^16^, MERS^17^, etc.). The occurrence of SSEs contributes to the speed and severity of an outbreak and also affects the development of disease management and prevention policies by health authorities. Human transmission has been documented for COVID-19^18^ and asymptomatic patients with SARS-CoV-2 infection can also transmit the virus^19^, leading to an urgent need to monitor SSEs, which could greatly aggravate the spread of COVID-19.

Person-to-person transmission patterns during the early stages of the COVID-19 pandemic have been inferred in this study. Due to the low sampling frequency, only a small number of direct transmission events (18 pairs of patients), with bidirectional probability for direct transmission > 0.5, were detected. Furthermore, the result from computational cross-validation on direct transmission events with high quality also indicated that the direct transmission events identified in our study were reliable. In addition, this result was robust to the uncertainty on phylogeny resulting from the low genetic diversity of viral genomes collected during the early stages of COVID-19 pandemic.

We also inferred the offspring distribution and, in particular, the dispersion parameter of this distribution. A recent study demonstrated that the dispersion parameter can be most accurately estimated when using phylogenetic data^20^. In this study, we used a phylogeny of SARS-CoV-2 to infer the dispersion parameter. The dispersion parameter was estimated as 0.23 (95% CI: 0.13–0.39), which was substantially smaller than 1, indicating the distribution of offspring was highly skewed or overdispersed due to the existence of SSEs^11^. This estimation was only based on the MCC tree, which represented the tree with the maximum sum of posterior clade probabilities in MCMC chains. However, we found that the uncertainty of the phylogeny would lead to an overestimation of the dispersion parameter (Fig. 3b). As smaller the dispersion parameter, the greater the heterogeneity of its distribution, overestimating the dispersion parameter would lead to underestimation of the degree of SSEs. Thus, it is reasonable to estimate the dispersion parameter by using MCC tree. The value of the dispersion parameter we estimated for COVID-19 was similar to previous estimates for Ebola virus disease (0.18, with 95% CI: 0.10–0.26)^21^ and SARS (0.16, with 90% CI: 0.11–0.64)^11^, indicating that SSEs also occurred in COVID-19 during the first 2 months. As the Chinese government has implemented a series of measures to avoid the flow and gathering of people after 23 January 2020^22^, it is less likely that SSEs would have happened after this date. Therefore, we speculated that the SSEs are likely to have occurred within the first month of the COVID-19 outbreak. Our findings suggested SSEs occurred early on, although this phenomenon has not been reported in previous studies^23,24^, which may be explained as follows. First, SSEs usually occur in densely populated and relatively closed spaces, such as hospitals and communities (like the Prince of Wales Hospital or the Amoy Gardens housing complex during the SARS epidemic)^10^. Due to the higher binding affinity to the receptor of human angiotensin I converting enzyme 2 (at least tenfold)^25^ and compatible aerosol and surface stability^26^, SARS-CoV-2 is likely to be transmitted more easily than SARS-CoV in humans. Combined with the finding that asymptomatic COVID-19 patients could also transmit virus^19^, it was more likely that spaces where super-spreading might occur could be more diverse, which could not be easily traced. Furthermore, the identification of SSEs was traditionally based on epidemiological tracing data, which depends on patient recall. However, it was difficult to trace person-to-person transmission using only epidemiological data, except for familial clusters. Second, the difference in the incubation period of different patients will also make it difficult to detect SSEs through epidemiological investigation during the early stages of the COVID-19 pandemic. The mean incubation period of COVID-19 was estimated to be 5.2 days and 95% of the distribution of incubation period was 12.5 days (95% CI 9.2–18)^23^, indicating that the incubation period varied greatly among patients. Compared with patients with a shorter incubation period, patients with a longer incubation period are more likely to infect more people during their incubation period. In particular, if the patients with a longer incubation period did not show obvious symptoms at the early phase of the COVID-19 pandemic, they will not be traced. In summary, the identification of SSEs during the early stages of the COVID-19 outbreak would be difficult based on epidemiological data, whereas our genomic approach circumvents this issue. In addition, several factors (including environment, human behaviors, mutations in human genome, etc.) can contribute to heterogeneities in the transmission of infectious diseases^10^. Future work should seek to identify these factors for COVID-19, so that we can tailor the disease management and prevention policies accordingly.

## Methods

As a wider spatial distribution of cases makes inference increasingly difficult as the pandemic expands, we focused on the study on early phase of COVID-19 outbreak. To strike a balance between the small amount of variation between viral genomes during the early stage of outbreak and sufficient variation to support this study, we defined the scope of the study to focus on the first 2 months of the outbreak. We only analyzed samples collected within the first 2 months (starting with the earliest sampling time until the next 60 days) in China. All SARS-CoV-2 genomes with high coverage from China were downloaded from GISAID. Only complete genomic sequences with exact collection date (accurate to days) were used in this study. Genomic sequences that are considered to contain many sequencing errors (https://virological.org/t/temporal-signal-and-the-evolutionary-rate-of-2019-n-cov-using-47-genomes-collected-by-feb-01-2020/379) were discarded from our analysis. In total, 208 SARS-CoV-2 genomic sequences were used in this study. The list of genomic sequences used in this study and their clinical information are provided in Supplementary Data 3 and 4, respectively.

After sequence alignment was performed with Mafft v7.310^27^, we trimmed the uncertain regions in 3′ and 5′ terminals (1–55 and 29,804–29,903 according to the 1-indexed coordinate of MN908947.3) and also masked 30 sites (Supplementary Table S1) that are highly homoplastic and have no phylogenetic signal as previous noted (https://virological.org/t/issues-with-sars-cov-2-sequencing-data/473), resulting in total genomic length of 29,718 nt. As recombination could impact the evolutionary signal, we searched for recombination events in these SARS-CoV-2 genomes using RDP4^28^. No evidence for recombination was found in our dataset. We used jModelTest v2.1.6^29^ to find the best substitution model according to the Bayesian Information Criterion. The best substitution model for our dataset was HKY + I. We then used the Bayesian MCMC approach implemented in BEAST v1.10.4^30^ to derive a dated phylogeny for SARS-CoV-2. Three replicate runs for each 100 million MCMC steps, sampling parameters, and trees every 10,000 steps. The estimation of the most appropriate combination of molecular clock and coalescent models for Bayesian phylogenetic analysis was determined using both path-sampling and stepping-stone models^31^. The best-fitting combination of prior molecular clock and coalescent model were an uncorrelated relaxed clock with log-normally distributed variation in rates among branches and Bayesian skyline tree prior (Supplementary Table S2). Tracer 1.7.1^32^ was then used to check the convergence of MCMC chain (effective sample size > 200) and to compute marginal posterior distributions of parameters, after discarding 10% of the MCMC chain as burn-in. The posterior distributions of phylogenies in the posterior tree space are shown in Supplementary Fig. 1. TreeAnnotator was used to summarize a MCC tree (Fig. 1a) from the posterior distribution of trees (after discarding 10% of the MCMC chain as burn-in). We also tested whether there was enough temporal molecular signal in this dataset. IQ-TREE 2.0.3^33^ was used to reconstruct the phylogeny under best substitution model (HKY + I) with 1000 ultrafast bootstrap replicates^34^. The relationship between root-to-tip divergence (from the phylogeny above) and sampling date for genomic data used in this study is also shown in Supplementary Fig. 2A using TempEst v1.5.3^35^. We also compared the prior and posterior distribution of parameters to determine the significance of the temporal signal in these genomic data. If there was a strong temporal signal in the dataset, the posterior distribution of parameters would be significantly shifted away from their prior distribution. As shown in Supplementary Fig. 2B, the prior and posterior distributions of tree heights were significantly different. These results confirm that there is sufficient temporal signal in our genomic data for reconstruct of a dated phylogeny.

As viral genomes were incompletely sampled and the pandemic is currently ongoing, TransPhylo v1.3.20^36^ was used to infer the transmission tree using the dated phylogeny generated above as input. The generation time (i.e., the time gap from infection to onward transmission, denoted as G) of COVID-19 was previously estimated as 7.5 ± 3.4 days^23^ and we used these values to compute the shape and scale parameter of a gamma distribution of G using the R package epitrix^37^. The distribution of sampling time (i.e., the time gap from infection to detection and sampling) was set equal to the distribution of generation time. We performed the TransPhylo analysis with 500,000 iterations simultaneously estimating the transmission tree, the proportion of sampling, the within-host coalescent time Neg, and the two parameters of the negative binomial offspring distribution (which represents the number of secondary cases caused by each infection). All results were generated after discarding 20% of the MCMC chains as burn-in. The MCMC mixing and convergence was assessed based on the effective sample size of each parameter (>200) and by visual examination of the MCMC traces (Supplementary Fig. 3 and Supplementary Data 5).

As the SARS-CoV-2 genomic sequences collected in the early stages of the pandemic were highly similar, it was difficult to obtain an exact phylogeny. Thus, we further tested whether the uncertainty in phylogeny affected the result. Ten dated phylogenetic trees were randomly selected from the MCMC chains (Supplementary Fig. 4) for TransPhylo analysis. The parameter setting was the same as above. These independent runs were performed with 500,000 iterations, with 20–30% of the MCMC chains discarded as burn-in (Supplementary Data 5). In addition, we performed cross-validation on the direct transmission pair with high probability. We randomly removed one patient in the top three pairs of patients with the highest bidirectional probability of direct transmission and then reconstructed the dated phylogeny to repeat the TransPhylo analysis. If all of the bidirectional probabilities of direct transmission between the retained patient from these three pairs and any other patient in this study are lower than 0.5, then the directed transmission events identified in our study are more likely to be reliable.

### Reporting summary

Further information on research design is available in the Nature Research Reporting Summary linked to this article.

## Supplementary information

Supplementary Information

Peer Review File

Descriptions of Additional Supplementary Files

Supplementary Data 1

Supplementary Data 2

Supplementary Data 3

Supplementary Data 4

Supplementary Data 5

Reporting Summary

## Data Availability

All data used in this manuscript are publicly available from GISAID. Accession numbers of genome sequences used in this study are listed in Supplementary Data 3.
