# Supplementary material for: Inference of person-to-person transmission of COVID-19 reveals hidden super-spreading events during the early outbreak phase
Source: Nat Commun. 2020 Oct 6;11:5006. doi: 10.1038/s41467-020-18836-4 (PMC7538999; doi:10.1038/s41467-020-18836-4)
Supplement: Supplementary file 1 — Supplementary Information [file 41467_2020_18836_MOESM1_ESM.pdf]

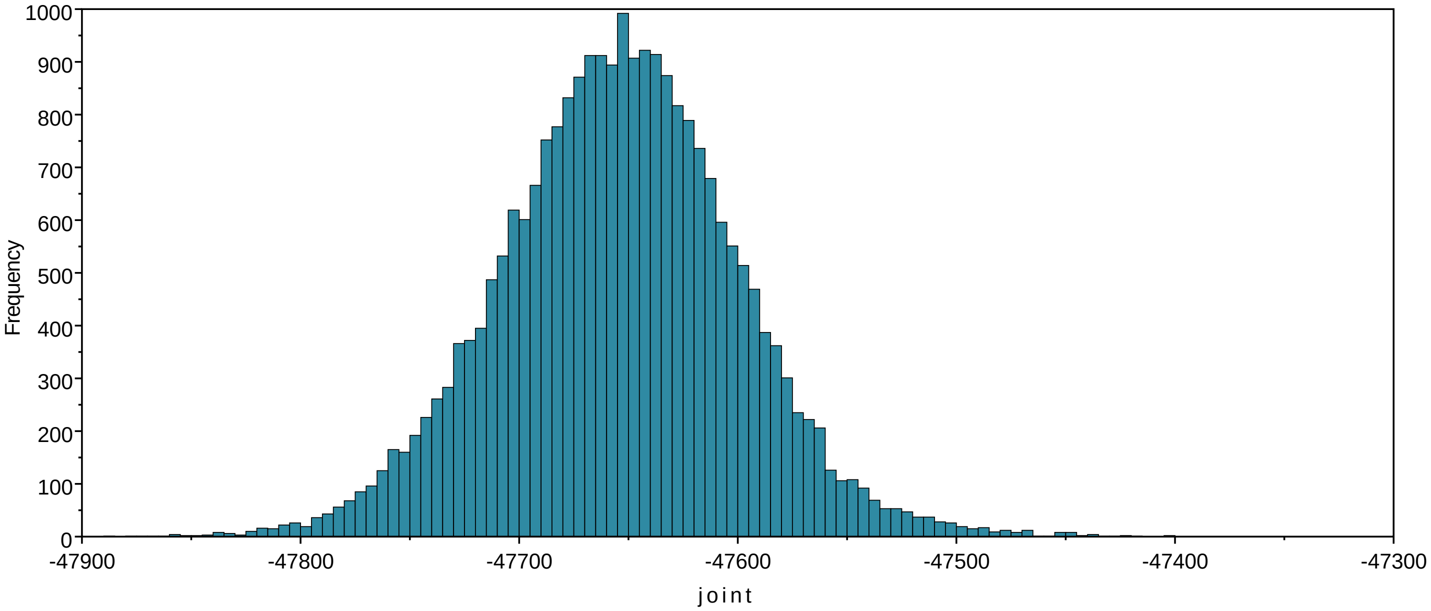

Supplementary Fig. 1. The posterior density distributions of the likelihood of phylogenies in the posterior tree space (merged by three replicate runs for each 100 million MCMC steps with after discarding 10% of the MCMC chain as burn-in).

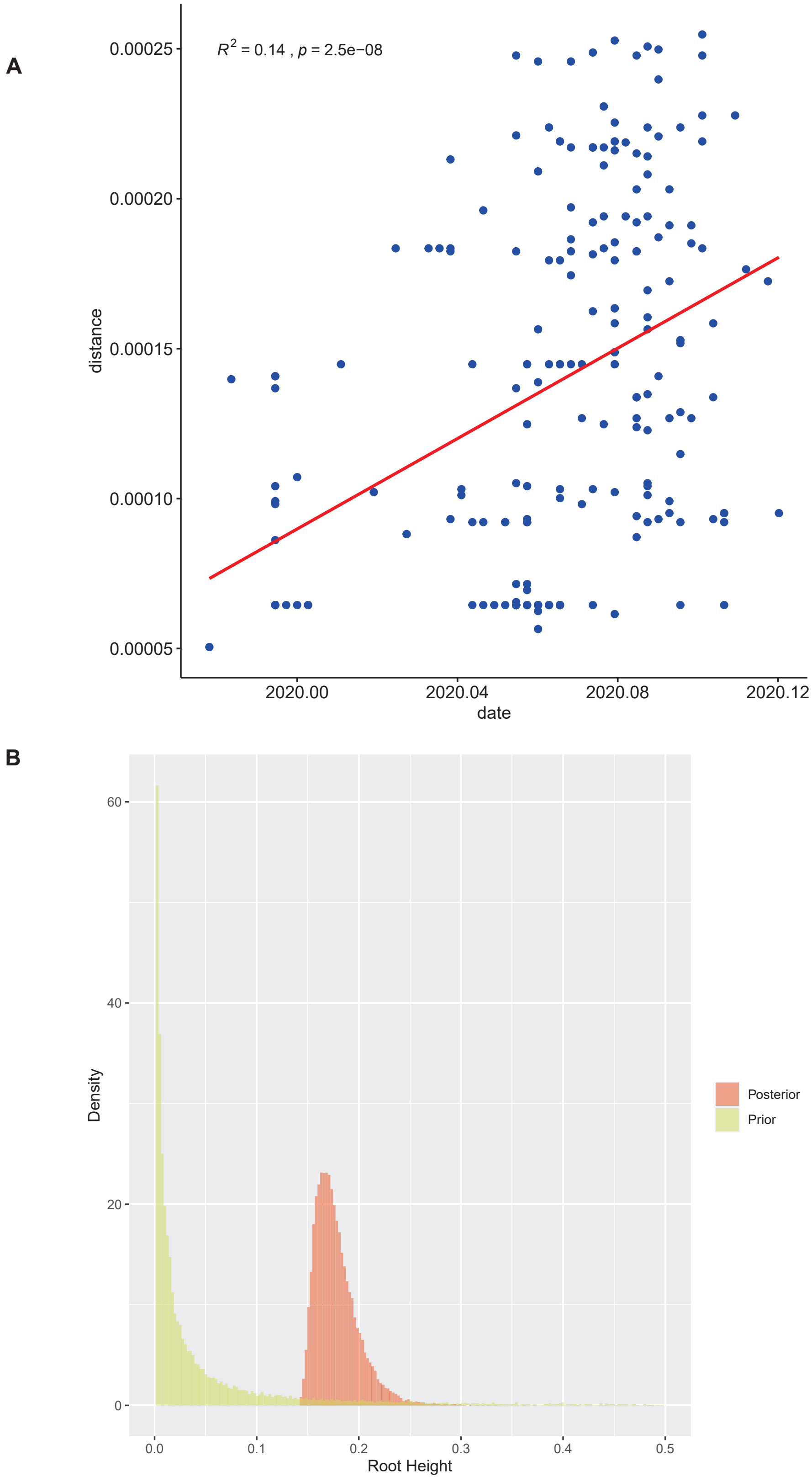

Supplementary Fig. 2. The temporal molecular evolutional signal in our datasets.

A. The correlation between root-to-tip divergence and sampling date for genomic data used in this study (n=208). A linear model was used to fit the data. Red line represents the regression line. *P* value was determined by two-sided Pearson correlation test.
B. The prior and posterior distribution of root heights for SARS-CoV-2 genomes in BEAST analysis.

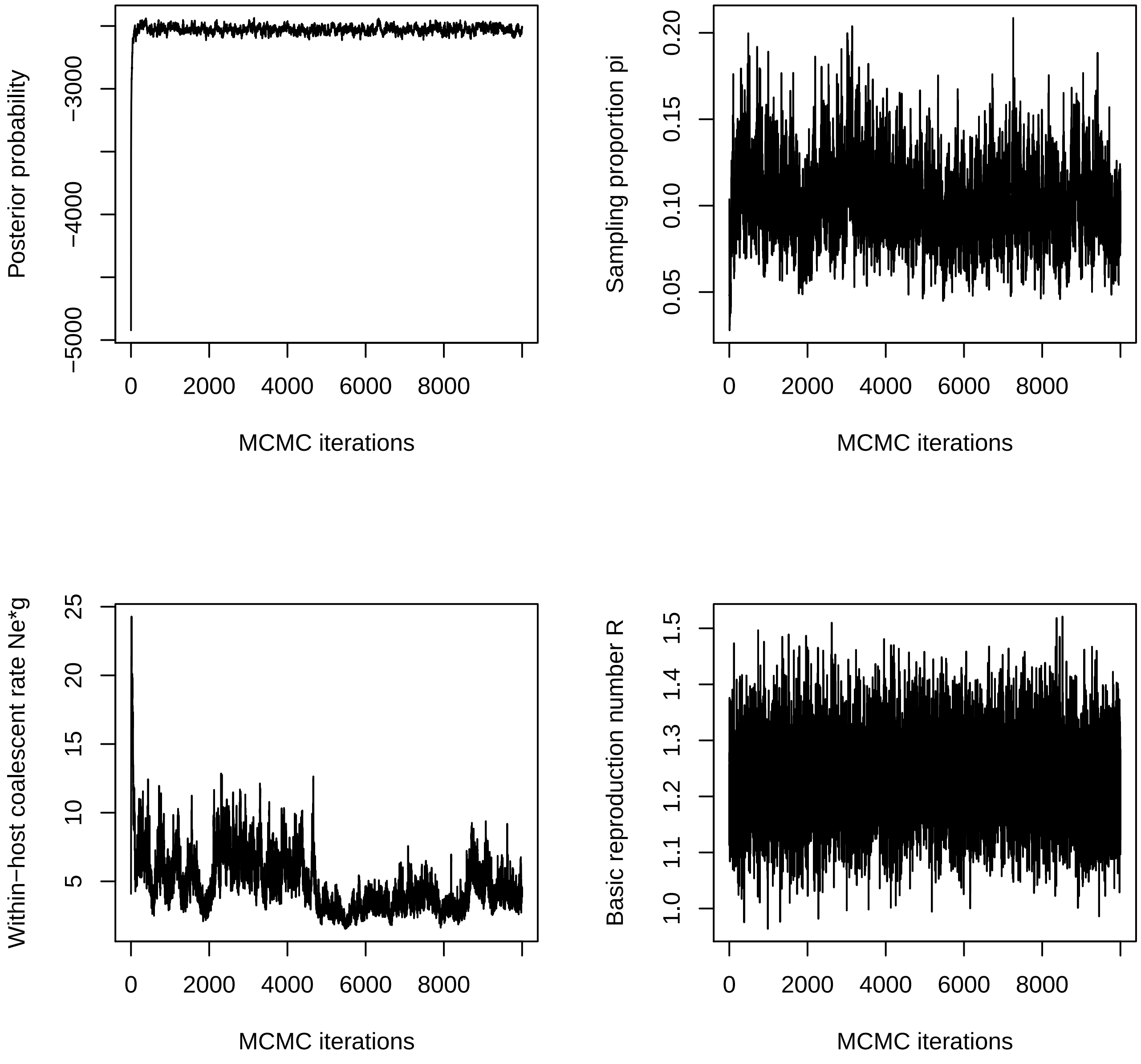

Supplementary Fig. 3. Trace plot of model parameters used in TransPhylo.

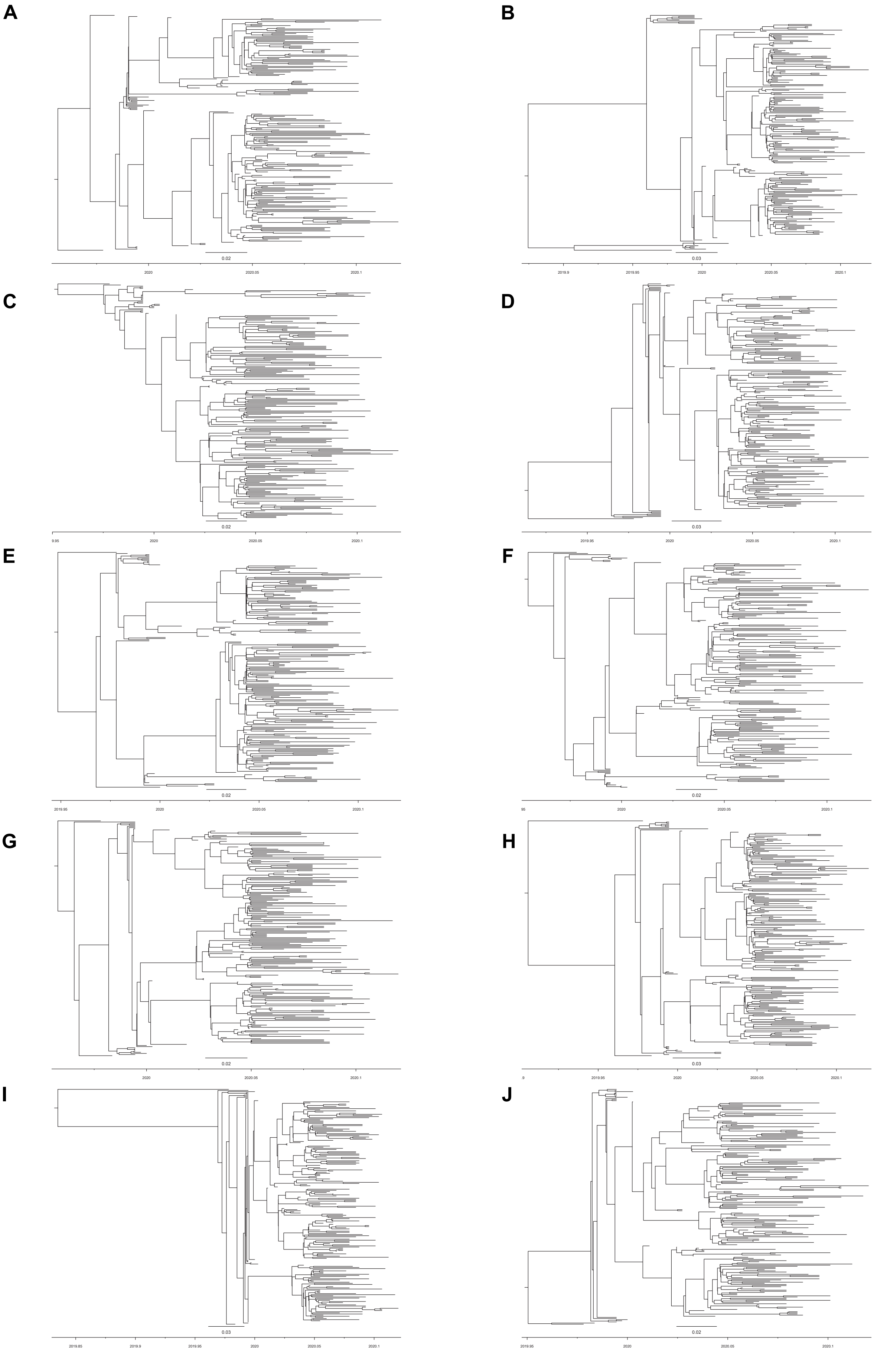

Supplementary Fig. 4. Topology of ten phylogenetic trees randomly selected from MCMC chains.

Supplementary Table 1. List of 30 masked sites in SARS-CoV-2 genome.

187
1059
2094
3037
3130
4050
6990
8022
10323
10741
11074
11083
13402
13408
14786
15324
19684
20148
21137
21575
24034
24378
25563
26144
26461
26681
28077
28826
28854
29700

Supplementary Table 2. Log-marginal likelihood estimates from model selection by using the path-sampling (PS) and stepping-stone (SS) approaches.

**Clock**	**Coalescent**	**path-sampling (PS)**	**stepping-stone (SS)**
Strict	Constant	-43454.00276	-43453.51454
Strict	Exponential	-43399.24855	-43399.13868
Strict	Skyline	-43383.61025	-43384.18542
UCLN*	Constant	-43448.40007	-43449.18935
UCLN*	Exponential	-43400.04203	-43400.25245
UCLN*	Skyline	-43383.32745	-43383.95451

*UCLN: uncorrelated relaxed clock with log-normal distribution
