# Supplementary material for: Inference of person-to-person transmission of COVID-19 reveals hidden super-spreading events during the early outbreak phase
Source: Nat Commun. 2020 Oct 6;11:5006. doi: 10.1038/s41467-020-18836-4 (PMC7538999; doi:10.1038/s41467-020-18836-4)
Supplement: Supplementary file 3 — Descriptions of Additional Supplementary Files [file 41467_2020_18836_MOESM3_ESM.pdf]

Descriptions of Additional Supplementary Files

Supplementary Data 1

Description: The bidirectional probability of direct transmission between any two patients.

Supplementary Data 2

Description: The number of intermediates between any two patients.

Supplementary Data 3

Description: The acknowledgement table of viral genomes used in this study.

Supplementary Data 4

Description: The clinical information of patients from which viral genomes used in this study were derived.

Supplementary Data 5

Description: The parameters of offspring distribution estimated by using different phylogeny.
