# Supplementary material for: Inference of person-to-person transmission of COVID-19 reveals hidden super-spreading events during the early outbreak phase
Source: Nat Commun. 2020 Oct 6;11:5006. doi: 10.1038/s41467-020-18836-4 (PMC7538999; doi:10.1038/s41467-020-18836-4)
Supplement: Supplementary file 9 — Reporting Summary [file 41467_2020_18836_MOESM9_ESM.pdf]

nature research

Corresponding author(s): Yuhai Bi

Last updated by author(s): Sep 3, 2020

Reporting Summary

Nature Research wishes to improve the reproducibility of the work that we publish. This form provides structure for consistency and transparency in reporting. For further information on Nature Research policies, seeAuthors & Referees and theEditorial Policy Checklist .

Statistics

For all statistical analyses, confirm that the following items are present in the figure legend, table legend, main text, or Methods section.

n/a	Confirmed	
☐	☒	The exact sample size (*n*) for each experimental group/condition, given as a discrete number and unit of measurement
☒	☐	A statement on whether measurements were taken from distinct samples or whether the same sample was measured repeatedly
☐	☒	The statistical test(s) used AND whether they are one- or two-sided<br>*Only common tests should be described solely by name; describe more complex techniques in the Methods section.*
☒	☐	A description of all covariates tested
☒	☐	A description of any assumptions or corrections, such as tests of normality and adjustment for multiple comparisons
☐	☒	A full description of the statistical parameters including central tendency (e.g. means) or other basic estimates (e.g. regression coefficient) AND variation (e.g. standard deviation) or associated estimates of uncertainty (e.g. confidence intervals)
☐	☒	For null hypothesis testing, the test statistic (e.g. *F*, *t*, *r*) with confidence intervals, effect sizes, degrees of freedom and *P* value noted<br>*Give P values as exact values whenever suitable.*
☐	☒	For Bayesian analysis, information on the choice of priors and Markov chain Monte Carlo settings
☒	☐	For hierarchical and complex designs, identification of the appropriate level for tests and full reporting of outcomes
☐	☒	Estimates of effect sizes (e.g. Cohen's *d*, Pearson's *r*), indicating how they were calculated

Our web collection on statistics for biologists contains articles on many of the points above.

Software and code

Policy information about availability of computer code

Data collection	Data were collected based on existing resources and no software was used
Data analysis	Sequence alignment: Mafft v7.310<br>Detection of recombination: RDP4<br>Model selection: jModelTest v2.1.6<br>Bayesian analysis: BEAST v1.10.4<br>Convergence detection of Bayesian analysis: Tracer v1.7.1<br>Reconstruction of transmission tree: TransPhylo v1.3.11<br>Reconstruction of phylogenetic tree: IQ-TREE 2.0.3<br>Detection of temporal signals in genomic data: TempEst v1.5.3<br>Calculating the shape and scale parameter of a gamma distribution of generation time: R package epitrix

For manuscripts utilizing custom algorithms or software that are central to the research but not yet described in published literature, software must be made available to editors/reviewers. We strongly encourage code deposition in a community repository (e.g. GitHub). See the Nature Research guidelines for submitting code & software for further information.

Data

Policy information about availability of data

All manuscripts must include a data availability statement. This statement should provide the following information, where applicable:

- Accession codes, unique identifiers, or web links for publicly available datasets
- A list of figures that have associated raw data
- A description of any restrictions on data availability

All data used in this manuscript are publicly available from GISAID. Accession number of genome sequences used in this study was list in Supplementary Data 1.

Field-specific reporting

Please select the one below that is the best fit for your research. If you are not sure, read the appropriate sections before making your selection.

- [x] Life sciences
- [ ] Behavioural & social sciences
- [ ] Ecological, evolutionary & environmental sciences

For a reference copy of the document with all sections, see nature.com/documents/nr-reporting-summary-flat.pdf

Life sciences study design

All studies must disclose on these points even when the disclosure is negative.

Sample size	No sample size calculation was performed before data collection and analysis, as we used all publicly available genomic data from China in GISAID during the first two month after the first confirmed case.
Data exclusions	We trimmed the uncertain regions in 3' and 5' terminals (1-55 and 29804-29903 according to the 1-indexed coordinate of MN908947.3) and also masked 30 sites (Table S1) that are highly homoplastic and have no phylogenetic signal as previous noted (https://virological.org/t/issues-with-sars-cov-2-sequencing-data/473), resulting in total genomic length of 29,718 nt.
Replication	Bayesian analysis was performed over three independent runs for each 100 million MCMC iterations. And all three replicates reached convergence (ESS>200 for all parameters).
Randomization	This is no relevant to the study as it is not a case/control study design.
Blinding	This is no relevant to the study as it is not a case/control study design.

Reporting for specific materials, systems and methods

We require information from authors about some types of materials, experimental systems and methods used in many studies. Here, indicate whether each material, system or method listed is relevant to your study. If you are not sure if a list item applies to your research, read the appropriate section before selecting a response.

Materials & experimental systems

n/a	Involved in the study	
x		Antibodies
x		Eukaryotic cell lines
x		Palaeontology
x		Animals and other organisms
x		Human research participants
x		Clinical data

Methods

n/a	Involved in the study	
x		ChIP-seq
x		Flow cytometry
x		MRI-based neuroimaging
