# Supplementary material for: Inference of person-to-person transmission of COVID-19 reveals hidden super-spreading events during the early outbreak phase
Source: Nat Commun. 2020 Oct 6;11:5006. doi: 10.1038/s41467-020-18836-4 (PMC7538999; doi:10.1038/s41467-020-18836-4)
Supplement: Supplementary file 2 — Peer Review File [file 41467_2020_18836_MOESM2_ESM.pdf]

Editorial Note: This manuscript has been previously reviewed at another journal that is not operating a transparent peer review scheme. This document only contains reviewer comments and rebuttal letters for versions considered at Nature Communications.

REVIEWERS' COMMENTS

Reviewer #1 (Remarks to the Author):

I think using the authors' choice to use the MCC tree for the point estimate of the dispersion parameter is appropriate, and it is good that they have expanded their dataset to increase their power to detect overdispersion. They now show the effect of the uncertainty in the phylogeny, which readers are free to interpret.

In short, they have addressed my remaining concern and I have no further issues with the study.

Stephen Schaffner

REVIEWERS' COMMENTS

Reviewer #1 (Remarks to the Author):

I think using the authors' choice to use the MCC tree for the point estimate of the dispersion parameter is appropriate, and it is good that they have expanded their dataset to increase their power to detect overdispersion. They now show the effect of the uncertainty in the phylogeny, which readers are free to interpret.

In short, they have addressed my remaining concern and I have no further issues with the study.

Stephen Schaffner

Response: We thank the reviewer again for the positive comment on our work.
